# If you want a quick kiss, make it count: How choice of syntactic construction affects event construal

**DOI:** 10.1016/j.jml.2016.12.001

**Published:** 2017-01-18

**Authors:** Eva Wittenberg, Roger Levy

**Affiliations:** a Department of Linguistics, University of California, San Diego, 9500 Gilman Drive, La Jolla, CA 92093-0108, United States; b Department of Brain and Cognitive Sciences, Massachusetts Institute of Technology, 77 Massachusetts Avenue, Cambridge, MA 02139-4307, United States

**Keywords:** Events, Lexical aspect, Light verb constructions, Mass–count distinction, Individuation, Linguistic framing

## Abstract

When we hear an event description, our mental construal is not only based on lexical items, but also on the message’s syntactic structure. This has been well-studied in the domains of causation, event participants, and object conceptualization. Less studied are the construals of temporality and numerosity as a function of syntax. We present a theory of how syntax affects the construal of event similarity and duration in a way that is systematically predictable from the interaction of mass/count syntax and verb semantics, and test these predictions in six studies. Punctive events in count syntax (*give a kiss*) and durative events in mass syntax (*give advice*) are construed as taking less time than in transitive frame (*kiss and advise*). Durative verbs in count syntax (*give a talk*), however, result in a semantic shift, orthogonal to duration estimates. These results demonstrate how syntactic and semantic structure together systematically affect event construal.

## Introduction

When people talk to each other about what happened, they usually don’t need to specify how long it took. Everybody knows from experience that a kiss lasts a few moments, a conference talk may carry on for about twenty minutes, and giving professional advice takes maybe half an hour, so there is typically no need to explicitly mention the duration. Duration is also usually not encoded grammatically. However, grammatical cues in event descriptions often significantly influence other aspects of event representations in listeners, such as causation, event structure, and the semantic roles of event participants (Fausey & Boroditsky, 2010; Johnson & Goldberg, 2013; Wittenberg & Snedeker, 2014). It would be all the more interesting, thus, if very subtle grammatical choices were to reliably affect how long listeners think an event takes.

In this article, we explore how encoding event descriptions in simple verbs (*to kiss, to advise*) versus count or mass noun light verb constructions (*to give a kiss, to give advice*) has repercussions on the temporal encoding of these events. Based on the fundamental observation that the reference properties of syntactic objects can change the reference properties of the whole predicate (Krifka, 1992), we predict that nominalizing an event can help dividing experience into countable units, influencing duration estimates in a way that is systematically predictable from the interaction of verb semantics and nominal syntax.

This hypothesis was inspired by a previous study on how events are individuated, depending on mass and count syntax. Barner, Wagner, and Snedeker (2008) found that using count syntax (*to do climbs*), but not mass syntax (*to do climbing*), affects how events are quantified; and that atomic,punctive events (*kissing, kicking*) are more readily quantified by counting over individual subevents (*kisses, kicks*) than non-atomic,durative events. This is in line with the Number Asymmetry hypothesis (Barner & Snedeker, 2006): whereas count syntax specifies individuation, mass syntax is underspecified.

If it is true that mass and count syntax contribute to event individuation, then we should expect predictable influences of mass or count syntax also on estimates of event duration. We distinguish between two types of events: Atomic, telic, mostly punctive events, like kissing or kicking; and non-atomic, atelic, mostly durative events, like talking or advising (Dowty, 1991; Vendler, 1957, see Footnote 1).

Punctive events are distinct from durative events not only in that they are conceptually short and bounded by a natural end point (telic), but also in that sentences in which they appear are often conventionally understood to describe several instances of the same punctive event, that is, they are understood iteratively (Barner et al., 2008; Kim & Kaiser, 2015; Paczynski, Jackendoff, & Kuperberg, 2014): For instance, you may find that *John kissed Mary* evokes the image of not one, but multiple kisses, each of which can be categorized as a subevent of kissing. Thus, punctive events can have a distinct substructure. Durative events, in contrast, are atelic and, they do not possess a distinct substructure, and they do not receive an iterative reading, even if the duration of the event is explicitly extended beyond a conventionally accepted time frame (cf. *Senator Cruz talked all night*).

Many of the aforementioned events, like *kiss*, advise or *talk*, can either be expressed as transitive verbs, or as so-called light verb constructions. In light verb constructions, the verb contributes little semantics beyond tense, number agreement, and aspect, while the meaning of the expression comes from the deverbal noun (Brugman, 2001; Butt, 2003; Butt, 2010; Jackendoff, 1974; Jespersen, 1954; Wiese, 2006). These light verb constructions appear either with count syntax, such as to *give a kiss* and *to give a talk*, or mass syntax, such as *to give advice*. Thus, light verb constructions offer us an opportunity to study the interaction of verb type and mass versus count syntax with an existing alternation, as opposed to unusual constructions such as *to do climbs* (Barner et al., 2008), or using novel lexical items (Wellwood, Hespos, & Rips, 2016): Light verb constructions, like *to give a kiss*, and their full verb counterparts, *like to kiss*, are in a relationship of syntactic alternation with minimal difference in meaning (Allerton, 2002; Glatz, 2006). In our study of punctive and durative events, we use light verb constructions with *give*, which is itself telic (Newman, 1996).

### The mass–count distinction and verbal aspect

Ever since Bach (1986), linguistic theory has been fascinated by the parallels between kinds of objects vs. substances on the one hand, and kinds of atomic vs. non-atomic events on the other hand (Casati & Varzi, 2008; Hale & Keyser, 1993; Harley, 2005; Jackendoff, 1991; Krifka, 1992; Quine, 1969; Rothstein, 2008; Verkuyl, 1972). One of the defining differences between objects and materials is that labels for objects denote atomic units, which cannot be partitioned arbitrarily: Only a whole apple, not a piece of an apple, can be described with the count noun *an apple*. A piece of an apple, on the other hand, will need to be further described with a quantifier or specific expression, such as *slice of an apple*, or *apple core*. Objects can also be individuated and counted (*three apples*). Materials, however, are non-atomic, and can be partitioned in an arbitrary fashion: a quart of applesauce can be divided into many portions, yet each individual portion still denotes *applesauce* (Bale & Barner, 2009; Rips & Hespos, 2015, and many others). Introducing individuability to mass nouns, however, is easily accomplished when they are quantized (*a bottle of wine, a quart of applesauce*; see Krifka, 1992; Wiese & Maling, 2005).

Events have the property of atomicity or non-atomicity, too: Some events are atomic, and some events are non-atomic. For example, if Mary kissed John, then she stopped just for a moment, and then started kissing him again, the post-interruption kiss would be a new event, even if the people and location are the same: an event of kissing is atomic in that it cannot be broken apart. (Note also that the character of the start and end points is constitutive of the event: if there is not contact between a set of lips and a surface, with a clearly defined onset and a clearly defined, voluntary or involuntary, offset, the term *kiss* does not apply.) This not true for all events (or processes, see Wellwood et al., 2016): If the president talked to a crowd, stopped for a moment, and started talking again, it could still be the same event of talking. Similarly, advising can be partitioned and spread over many advising sessions, but the overarching event of advising is the same, as long as there is some degree of spatial or character continuity (Anderson, Garrod, & Sanford, 1983; Magliano & Zacks, 2011; Zacks & Tversky, 2001). Talking and advising are thus non-atomic: they can thus be broken up and still count as the same talking and advising events.

The atomicity and non-atomicity of events is highly correlated with notions of telicity, boundedness, and aspect in verbs or predicates, as well as the distinction between events and processes in some approaches.^1^ For the purpose of this article, atomicity as described above will be the defining criterion for an event to be classified as either punctive (atomic, e.g., *kiss*) or durative (non-atomic, e.g., *talk, advise*), even though the overlap of terminologies is not perfect.

In this study, we focus on the interaction of count and mass syntax with punctive and durative events in a syntactic ditransitive frame provided by the verb give, such as *Mary gave Douglas a kiss, the professor gave her student advice*, or *the president gave a talk to the audience*. Given the parallels between between mass/count syntax and verbal aspect, and given the telic verb *give*, we expect count and mass syntax to interact differently with punctive and durative event nouns: Count syntax with a punctive deverbal noun like *giving a kiss* should pick out one single instance of *kissing*; mass syntax with a durative deverbal noun like *giving advice* should carve out a portion of *advising*. If this is true, then the grammar conveys a subtle difference in event duration between the simple transitive verb and the light verb construction, for any given event: the light verb construction communicates an event that lasts a shorter time.

For durative events in count syntax in combination with the telic light verb *give*, however, the predictions are not as straightforward. Just consider what happens when one packages substances like *beer, glass, string, stone* or *iron* into count syntax: In some cases, the count noun denotes portions (of arbitrary size) of the substance (*a string, a stone, a beer*), but in other cases, the count noun phrase happens to encode objects or units that are related to the mass noun, yet in an arbitrary way, such as in *a glass or an iron*. Crucially, the resulting denotation for mass nouns in count syntax is variable, and each case conveys aspects of meaning that cannot be predicted from the intrinsic structure of the underlying substance (Gordon, 1985; Srinivasan & Rabagliati, 2015).

So what should we expect in the case of durative verbs entering count syntax, like *talk* in *give a talk*? If the analogy between durative verbs and substance nouns really holds, one would predict a certain degree of conventionalization, similar to the *some glass* → *a glass case*: Core parts of a given durative event would remain the same, but the event type would shift in meaning from verb to count noun construction, possibly closing a lexical gap in doing so (Allerton, 2002; Glatz, 2006; Grimshaw & Mester, 1988; Miyagawa, 1989). For example, there is a strong intuition that *giving a talk*, albeit still retaining the core meaning of utterance production, is conceptually further from *talking* than *giving a kiss* is from *kissing*.

But then, if it is true that durative events in count syntax undergo a conceptual shift in event kind, predictions about event duration are up in the air, since the change induced by count syntax would be orthogonal to changes in temporal conceptualization. In this article we explore both sides: Whether event duration estimates are modulated by the introduction of mass and count syntax, and whether there is an effect on how similar events are judged as depending on the syntactic construction. In the next section, we will discuss the link between event representation and linguistic encoding in more detail.

### Event construal via linguistic encoding

How linguistic framing influences people’s event conceptualization, memory, and recall has long been a topic of interest in science, such as in behavioral economics (Halkjelsvik, Jørgensen, & Teigen, 2011; Kahneman & Tversky, 1977; Kruger & Evans, 2004; Roy, Christenfeld, & McKenzie, 2005), criminal justice (Boltz, 1995; Loftus, Schooler, Boone, & Kline, 1987; Macar, Grondin, & Casini, 1994; Tse, Intriligator, Rivest, & Cavanagh, 2004), and cognitive science (Madden & Zwaan, 2003; Magliano & Schleich, 2000). These studies, however, were mainly concerned with behavioral or memory consequences of linguistic encoding accompanying visual scenes, and less with representational or grammatical issues.

In psycholinguistics, using grammatical alternations to study their influence on event construal started with Gropen, Pinker, Hollander, and Goldberg (1991), who found that subtle changes to event structure affected which form of the locative alternation people used in production (*cover a surface with marbles or dropping marbles onto a surface*). A later study confirmed the intuition that syntactically omitting agents from an event description reduces how much blame is assigned to them (Fausey & Boroditsky, 2010). Directly related to the constructions used in this article, we know that using a light verb construction with give influences the construal of thematic roles (Wittenberg, Khan, & Snedeker, submitted for publication; Wittenberg & Snedeker, 2014. And finally, there is evidence from a production study that the naturalness of event divisions predicts the choice of mass or count syntax (Wellwood et al., 2016).

The question of whether differences in event descriptions cause differences in duration estimates is less well studied. So far, there is a small experimental literature showing that verbal aspect or the verb itself influence duration estimates. For example, human locomotion events seen on video are remembered as taking longer when they are described as walking events than when they are described as running events (Burt & Popple, 1996); and a Dutch study has shown that describing a short event in progressive verb aspect (*is kissing*) makes people think that the kiss took longer than if simple present is used (Flecken & Gerwien, 2013). Pedersen & Wright (2002), in contrast, found only small effects of event descriptions on the duration estimates; however, their manipulation was on writing style and purposefully not as tightly controlled for semantic and syntactic factors as other studies. Importantly, Coll-Florit & Gennari (2011, Study 4) found that event duration estimates are tightly linked to aspectual nature of verbs.

Thus, there is some evidence that linguistic choices influence the way people think about the temporal dimensions of events. Yet, it is not surprising that one should find this influence by grammatical means that by default operate in the temporal dimension, like aspect, or by choosing lexical items according to the speed of an action that they express. The alternation between transitive verbs and their light verb construction counterparts, however, affords a way to look beyond the more obvious aspects of how syn tactic and semantic regularities work together to create a rich, full, and detailed representation of an event.

How could this interaction work? Most current theoretical approaches have accounted for the fact that some lexical items can appear both as nouns and verbs with the stipulation that prior to lexical insertion, their grammatical status is neutral. That means, a word like *kiss* only acquires its grammatical category upon insertion into a syntactic tree; either way, regardless of whether it is inserted as a noun (such as *He gave her a kiss*) or a verb (*He kissed her*), it conceptually refers to the same kind of event (Barner & Bale, 2002; Halle & Marantz, 1994).

Taking this as a starting point, we should be able to observe a systematic interaction between syntax, the lexicon, and event construal. We assume that syntactic, conceptual, lexical, and phonological structure interact and predict upcoming features and structures every step of the way (Garrod & Pickering, 2003; Jackendoff, 2002; Jackendoff, 2007; Levy, 2008; Martin, 2016).^2^ This architecture of the linguistic system allows for an interaction between representations on the levels of syntax (mass and count syntax), semantics (the telicity of *give*), and event knowledge (how long kissing usually takes). Other approaches (Goldberg, 1995; Goldberg, 2003) explicitly allow for different meaning shades of grammatical constructions; presumably, the distinct meaning of a ditransitive construction as a telic event would be even more straightforwardly predicted from this perspective. Crucially, both of these models would predict differences in duration estimates due to the interaction of verbal and nominal semantics and syntax.

### Key theoretical predictions and the current studies

**Fig. 1.**
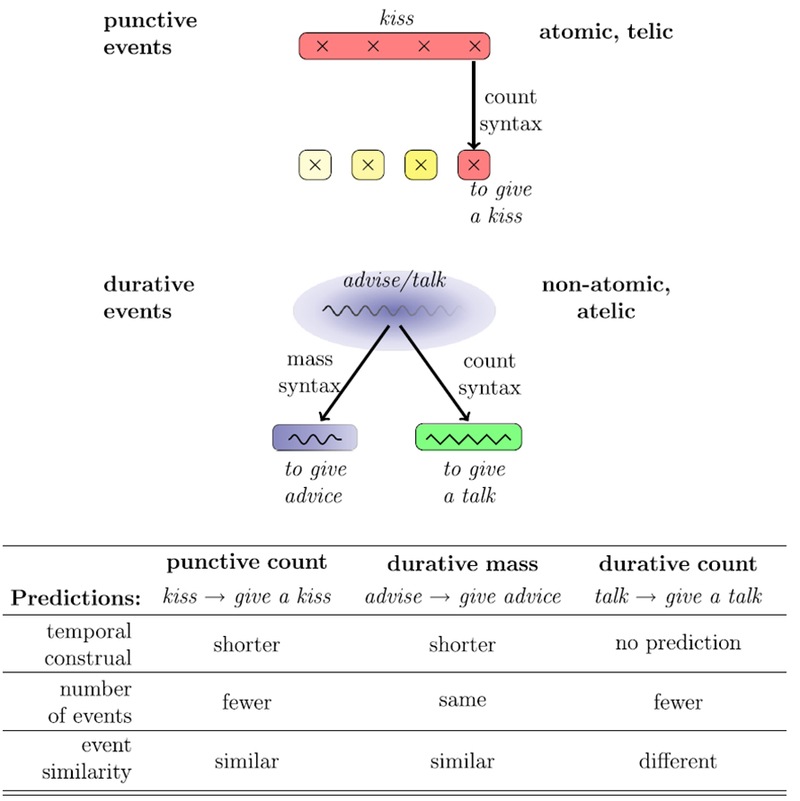
Predictions of how mass versus count syntax interacts with verb semantics, with regards to event duration construals, number of events, and event similarity.

The current studies investigate whether describing an event with mass or count syntax, as opposed to a simple transitive verb, affects the construal of event duration, event similarity, and event repetitions in a comprehender. Fig. 1 shows a graphical representation of our theory.

Starting from the top of Fig. 1, we look at punctive events like *kissing*. Previous studies have shown that punctive events are often construed as occurring more than once (Barner et al., 2008; Kim & Kaiser, 2015; Paczynski et al., 2014), e.g., a comprehender might hear *John kissed Mary* and imagine more than one kiss. In Fig. 1, this is represented by four crosses, which are distinctive, atomic subevents within one iterated,bounded kissing event. Count syntax, according to our theory, should encourage event individuation in iterative events: In the case of our example, *giving a kiss* describes only one atom of a kiss.This in turn should have repercussions in the construal of event temporality, leading to conceptualizations of shorter event duration. Another prediction is that punctive events in count syntax will be construed as consisting of fewer event iterations than in transitive syntax.

The bottom of Fig. 1 visualizes our predictions for durative events. Durative events, represented by the continuous snake line, have no set endpoint (and in many cases, no set starting point either). They are also non-atomic; for example, *advising* is a process that consists of many points in time, but it is hard to conceptually delimit when advising starts and ends based on these time points. However, it is possible to carve out a particular portion of this process, and we claim that this is linguistically done when the event appears in mass syntax: *to give advice* refers to a chunk of advising whose boundaries are limited (although not quite as strictly as in the case of *to give a kiss*). In terms of event counts, the predictions are weaker than for the punctive event counts, since mass syntax does not aid in event individuation (although the telicity of the verb *give* might).

When durative events occur in count syntax, such as in *to give a talk* (bottom right on Fig. 1), we hypothesize that, analogous to the cases of using mass nouns in count syntax (*glass, iron versus* *a* *glass*, *an* *iron*), there are arbitrary and unpredictable changes in the kinds of events these nouns then describe. For example, *giving a talk* still retains a sense of utterance production, but in a very different context and with different event parameters (this difference is represented by a change in color, and the zigzag line, in Fig. 1). This creates another prediction: *Talking and giving a talk* should be conceptually less closely related than punctive count and durative mass pairs. In terms of event counts, we predict that count syntax form will help event individuation and possibly lead to a reduction in event counts. Crucially, both event similarity and event count differences would operate entirely orthogonally from the construal of event duration.

We present six experiments that test these predictions. To test the claim that the construal of event duration is predictable from the interaction of mass versus count syntax, and verb semantics, we present two studies that elicited open estimates of event duration (Experiment 1a and 1b); in order to ensure that the estimates obtained in Experiments 1a and 1b are not due to task difficulty, we then present a temporal categorization experiment, in which participants categorized event descriptions into predefined time bins (Experiment 2). Then we present two studies that establish whether using count or mass syntax affects how many events people imagine: Experiments 3a and 3b investigate whether the determiner “a” in ditransitive count syntax picks out one particular instance of an event, which would expound shorter temporal estimates in count syntax. Finally we investigate whether describing durative events in count syntax indeed leads to conceptual shifts in event type, which would explain the inconsistent pattern observed for durative events (Experiment 4).

### Stimuli used for all studies

The verbs we used cluster together in a number of semantic and syntactic factors: For example, all events in the “punctive” category are atomic, short and in themselves telic. They are, incidentally, also all contact verbs. In a light verb construction, they appear in count syntax. The events in the “durative count” category are all non-atomic, of medium duration, and atelic. Incidentally, they are also all actions of utterance towards others. When appearing in a light verb construction, they do so with count syntax. Their semantic common denominator may be best captured as social actions with the direct object as beneficiary. When appearing in a light verb construction, they take on mass syntax. Above and beyond other semantic aspects, however, atomicity of the verbs is the most important factor for the purpose of this article, and we used the notions “punctive” and “durative” to distinguish between those categories of events.

We used punctive verbs (*kiss*) and durative verbs (*advise, talk*) either in a transitive frame (*After their first date, John kissed Mary*) or in a ditransitive light verb construction with the telic verb *give* (*After their first date, John gave a kiss to Mary*) such as in Table 1 (see Appendix A for a full list of stimuli).^3^ The ditransitive frame introduces a distinction between count syntax (*give a kiss/talk*) and mass syntax (*give advice*). We expected count syntax to force event individuation in punctive verbs, such that, when asked about event duration, people should judge the same event to be shorter in the ditransitive than in the transitive frame. For durative verbs, we predicted the same pattern for mass syntax, albeit to a lesser degree, because only the light verb *give* would encourage telicity. For durative events that enter count syntax, we predicted a different pattern: Since there are no distinctive subevents that can be counted, applying count syntax to durative verbs should not lead to differences in duration. Instead, it should open the door to different event construals, orthogonal to changes in temporal structure (see Fig. 1 for an overview of predictions).

Of the twenty experimental items, seven encoded punctive events, e.g. *kissing/giving* a kiss, six were durative events and could be used with count syntax, e.g. *talking/giving a talk*, and seven encoded durative events that could be used with mass syntax, e.g. *advising/giving advice*.^4^ Note that in this last category, the noun describing the action (*advice*) was not preceded by a determiner. None of the light verb constructions that we used can alternate between mass and count syntax (e.g. to give *(a) kiss; to give (*an) advice). In short, there were three different verb alternations: punctive transitive vs. ditransitive with count syntax (punctive count); durative transitive vs. ditransitive count syntax (durative count); and durative transitive vs. ditransitive mass syntax (durative mass).

In all studies except for Experiment 4, the items were embedded in a sentence context. All sentences used the simple past. Each sentence included a temporal or local adjunct phrase, to encourage non-repetitive readings (e.g. *The professor advised her student on his paper* *yesterday*). In addition to the experimental items, we also created 27 filler sentences.

## Experiment 1a: Open estimates of event duration

In Experiment 1a, we asked participants to rate how long events took. The events were described by simple transitive or light ditransitive constructions, and we were interested in whether the predictions displayed in Fig. 1 would be confirmed. If so, we expect punctive events like kissing to be estimated as taking less time in the ditransitive construction (*giving a kiss*). We should also observe the same trend for durative events when they occur in mass syntax (*advising – giving advice*), but not durative events in count syntax (*talking – giving a talk*). Experiment 1b served as a replication, with event category as a between-subjects factor.

### Methods

#### Participants

We recruited 100 unique individuals on Amazon Mechanical Turk, an online crowd-sourcing tool. Mechanical Turk allows access to a large number of study participants, who participate anonymously for reasonable compensation, in our case for about $6 an hour (Buhrmester, Kwang, & Gosling, 2011; Crump, McDonnell, & Gureckis, 2013). Our participants had IP addresses within the United States and were self-reported native speakers of English.

#### Stimuli

We created two lists out of the sentences described above and distributed the experimental items across them with a fully within-subjects Latin-square design, such that each participant saw each sentence in only one of the two constructional forms (transitive, or ditransitive light verb). The fillers were the same across lists.

#### Procedure

Participants read each sentence and then estimated how long the event described in the sentence probably took. The exact instructions can be found under https://github.com/ewittenberg/QuickKissing.

For each item, participants were able to enter their estimated event duration in a set of three text boxes, one for hours, one for minutes, and one for seconds, e.g., a participant could respond “1 h(s), 13 min(s), 7 s(s)”. Empty boxes were treated as a response of zero for that unit of time. Completing the study took about 17 min on average.

#### Results

**Fig. 2.**
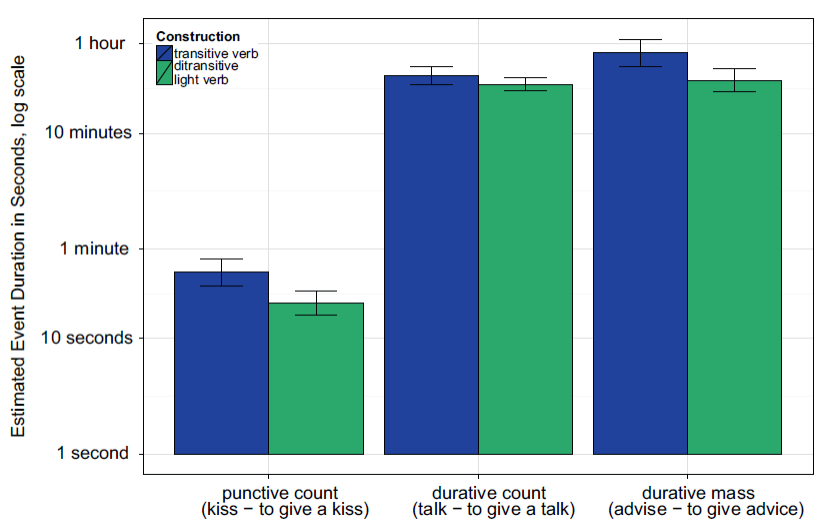
Experiment 1a: Duration estimates per item pair. The y-axis is represented in log scale, but labeled with reader-friendly time estimates for convenience.

Responses in which the estimated duration was zero were discarded. This affected less than .1% of the data. Since effects of grammatical structure on event duration would be likely to operate proportionally to intrinsic event duration, we transformed all responses to log-seconds for purposes of data summarization and analysis. Fig. 2 shows the pattern of results, with responses back-transformed to hours, minutes, and seconds, for convenience of interpretation. In all figures in the article, bar plots show means and Standard Errors of by-subject means unless otherwise stated.

For punctive count events, using a ditransitive light verb construction instead of the transitive verb cut the time estimates in half, from about 40 to about 20 s. For durative count items, the effect was smaller (transitive *μ* = 31 min, ditransitive *μ* = 27 min), but in durative mass items, it was even stronger than for punctive count events (transitive *μ* = 50 min, ditransitive *μ* = 29 min).

For data from this and all following experiments (with the exception of Experiment 4, which has a different design), we conducted two types of statistical analysis. The first is a 2 × 3 ANOVA-style analysis of the main effect of construction (transitive or ditransitive light verb), the omnibus main effect of event category (punctive count, durative count, and durative mass), and the omnibus interaction between the two. The second is a set of planned pairwise tests of the effect of construction within each event category. The reason for this latter set of planned tests is that the strength of evidence for an effect of construction for each event category is relevant to assessing the overall support of the data for our main hypothesis regarding effects of syntactic construction on event construal.

In all analyses we used mixed-effects regression models (Baayen, Davidson, & Bates, 2008; in this experiment, linear mixed-effects models) with R’s lme4 package (Bates et al., 2014), using maximal random effects structure justified by the design (Barr, Levy, Scheepers, & Tily, 2013; where noted, random correlation parameters were dropped to ensure model convergence) and computing *p*-values through likelihood-ratio tests between models differing only in the presence or absence of the fixed-effect parameter(s) being tested. We used Helmert coding for both fixed-effects predictors, grouping punctive count and durative mass items together as one Helmert contrast pair, and their average contrasted with durative count items as the second Helmert contrast pair.^5^

The top half of Table 2 shows the results of the 2 × 3 ANOVA-style analyses (these analyses involved random by-participant intercepts and slopes for all fixed effects and random by-item intercepts and slopes for construction, with all random correlation parameters removed). We see significant main effects of construction and event category; the interaction does not reach statistical significance.

The bottom half of Table 2 shows results of the planned pairwise tests within each event category (with random intercepts and slopes for both participants and items; no random correlation parameters needed to be omitted). Whenever punctive events appear in a count light verb construction with *give* (ditransitive frame, such as *to give a kiss*), they are estimated to take less time than when they appear in a simple transitive (*to kiss*). This effect was marginally significant. For durative events in mass syntax (*to give advice – to advise*), the same pattern was statistically significant; the numeric pattern for durative events in count syntax is far from statistically significant (*to give a talk – to talk*).

#### Replication: Experiment 1b

We replicated this study with 300 participants on Amazon Mechanical Turk by making event type (punctive count, durative count or durative mass) a between-subjects factor, reasoning that thinking about events on vastly different time scales might wash out sharper judgments. For example, the literature on prospective time estimation shows that in events lasting more than a few moments, people count internally to “track time”, while for very short events, this strategy is not used (Grondin, Meilleur-Wells, & Lachance, 1999; Zakay & Block, 1995). Similarly, short events like kissing or hugging might be imagined by conjuring up an image of this event, while for longer events like talking or advising, abstract world knowledge might be employed to estimate duration. Experiment 1b served to exclude this potential mix of strategies in temporal estimation. The exact instructions can be found under https://github.com/ewittenberg/QuickKissing.

**Fig. 3.**
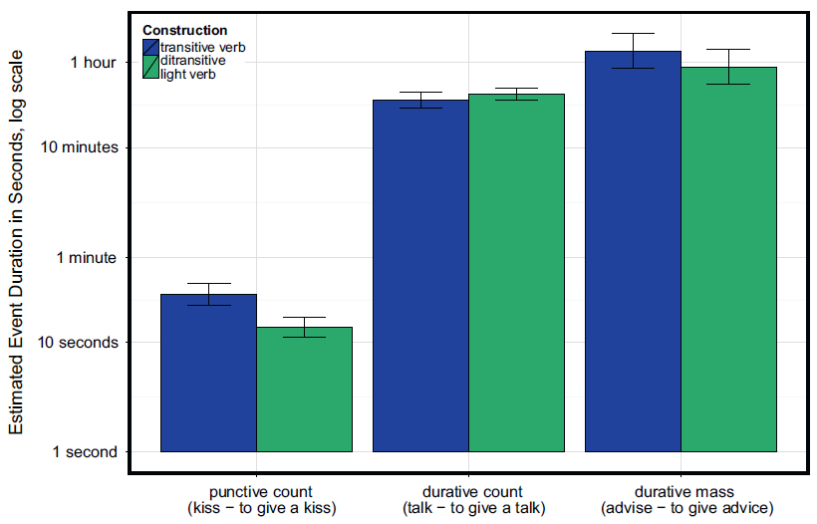
Experiment 1b (replication with event category as between-subjects factor): Duration estimates per item pair. The y-axis is represented in log scale, but labeled with reader-friendly time estimates for convenience.

Fig. 3 shows the pattern of results; as in Experiment 1a, punctive count item pairs were estimated to take roughly half as long when they were presented in ditransitive light verb frames (transitive *μ* = 27 s, ditransitive *μ* = 14 s). Durative count items showed no trace of an effect of syntactic construction, with mean estimates of 27 min in the transitive frame and 31 min in the ditransitive frame. Durative mass items showed the same numeric pattern as punctive count items, with mean estimates of 76 min in the transitive frame and 54 min in the ditransitive frame, although this difference was not statistically significant.

Table 3 shows the results of the regression analyses, conducted identically as those in Experiment 1a except that random by-subjects slopes for event category and its interaction with construction were excluded, since event category was a between-subjects manipulation. The top half of Table 3 shows the results of the ANOVA-style analyses likelihood-ratio tests (since event category was both between subjects and between items, random by-subject slope for event category, or the random interaction, were not needed). Both main effects of construction and event category, and their interaction, were significant.

The planned pairwise comparison results are shown in the bottom half of Table 3 (here, random correlation parameters did not need to be removed). As in Experiment 1a, whenever punctive events appear in a count light verb construction with give (ditransitive frame, such *asto give a kiss*), they are estimated to take less time than when they appear in a simple transitive (*to kiss*). The effect was not significant for durative events with mass syntax (*to give advice – to advise*), or for durative events with count syntax (*to give a talk – to talk*).

#### Discussion of Experiments 1a and 1b

As described above, the theory we proposed of how linguistic encoding and event type interact in event construal strongly predicted an effect of construction (transitive versus light-verb) on inferred event duration for punctive events whose light-verb encoding involves count syntax (*kissing vs. giving a kiss*): light-verb syntax should shorten inferred event durations. This prediction was borne out in both Experiments 1a and 1b. The theory predicted no systematic effect on inferred event duration for durative events encoded with count syntax, as this encoding requires more substantial, conventionalized shifts (*talk vs. give a talk*). This prediction was also borne out: there was no trace of a systematic effect of construction on inferred event duration across Experiments 1a and 1b. We suggested that the theory’s predictions were less clear for durative events encoded with mass syntax (*advise vs. give advice*), and indeed our results across the two experiments were less clear. We found a significant shortening effect of light-verb syntax in Experiment 1a; in Experiment 1b, the same numeric pattern was evident but did not reach significance.

As a methodological note, one might notice that the duration estimates seemed fairly high. We know from previous studies that estimating the duration of an event is often influenced by its pleasantness or desirability (Kahneman & Tversky, 1977; Roy et al., 2005). Although the numerical values of the estimated event durations are not crucial here, since we are only interested in the difference in estimates that is attributable to syntactic structure, it is conceivable that our participants’ estimates were influenced by factors not due to the grammatical construction alone. For this reason, we decided to replicate this study as a categorization task with predefined time windows as answer options. The predefined time windows were based on previous answers, to validate the results obtained in Experiment 1a and 1b.

## Experiment 2: Categorizations of event duration

This experiment gave participants the opportunity to estimate event durations without needing to come up with their own time estimates, and instead being able to select among predefined time bins for each individual event.

### Method

#### Participants

We recruited 80 self-reported native speakers of English on Amazon Mechanical Turk with IP addresses within the United States.

#### Stimuli

We used the same sentences as in the previous studies. Participants read each sentence and then had to categorize the event for duration by clicking one of four options. These options were created from the quartiles of the empirical distribution of estimated durations of each item pair in Experiment 1b; thus, every item had different answer options, as in Examples (1) and (2):

1. *Laughing nastily, the thug kicked the victim*. How long did this take?
  - up to 5 seconds,
  - between 5 seconds and 13 seconds,
  - between 13 seconds and 10 minutes,
  - more than 10 minutes.
2. *The professor advised her student on his paper yesterday*. How long did this take?
  - up to 25 minutes,
  - between 25 minutes and 1 hour,
  - between 1 hour and 2 weeks,
  - more than 2 weeks.

The same lists as in Experiment 1a were used.

#### Results

**Fig. 4.**
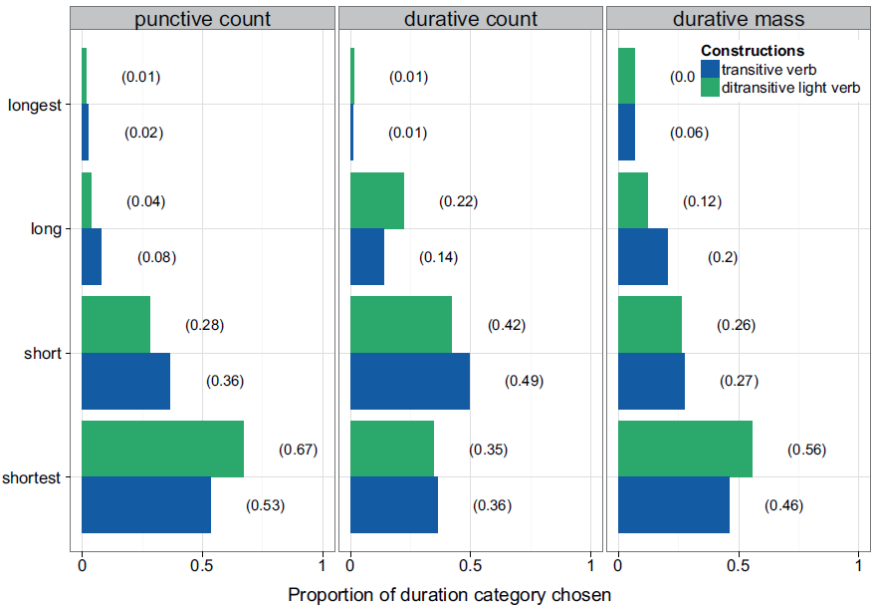
Experiment 2: Proportion of duration categories chosen, per event type and construction.

Fig. 4 shows the proportional distribution of categorizations into the four time bins, and the proportion of counts in each category, depending on event category and grammatical construction. In general, we observed a tendency to categorize all events into the shorter time bins. More theoretically crucial, light verb constructions were categorized as being shortest.

To assess the strength of evidence for the light verb construction shifting responses systematically toward the shorter quartiles (and the strength of evidence for any interaction of such an effect with event category), we used a mixed-effects cumulative logit model using R’s ordinal package (Christensen, 2015). For ordered response categories (in our case, four bins ranging from shortest to longest), a mixed-effects cumulative logit model specifies response probabilities for a given data point as a function of predictor variables. Instead of the intercept, ordered logit models provide a set of threshold parameters, which describe the boundaries from one bin to the next, and the probability of being drawn from one particular bin is estimated by the linear predictors with the inverse logit function (see Appendix B for an in-depth explanation of the mathematical underpinnings). The predictor variables in our model were construction (transitive or ditransitive), event category (punctive count, durative count, or durative mass), and their interaction. We Helmert-coded predictors and used maximal random effects structure as in Experiment 1a. Under this coding, construction is coded with transitive=−1, ditransitive (light verb)=1. Response categories were coded 1 to 4 in order of increasing duration. For this model and predictor coding, if the light verb construction yields systematically shorter inferred event durations than the transitive construction, it should manifest as a significantly *negative* parameter estimate for the fixed effect of construction.

Table 4 shows the statistical results of the categorization task. The first three rows indicate the threshold coefficients from one bin into the next. The middle part of the table shows the regression coefficient for construction – the –0.195 value indicating that the ditransitive construction is associated with shorter event durations – and the results of the likelihood-ratio tests for the main effects and their interaction, using likelihood-ratio tests as in Experiments 1a and 1b. Construction had a significant effect on duration estimates in the model comparison, and the interaction between construction and event category was marginally significant, but the main effect of event category was not. The lack of a main effect of event category is reassuring given that the time bins were constructed based on duration estimates from Experiment 1a.

Finally, the bottom part of Table 4 displays the results of analyses of the main effect of construction within each event category. For punctive count pairs, there was a significant difference in categorizations depending on construction. For durative count and durative mass items, this difference was not significant; however, a look at the *β*-estimates tells us that the effect of construction went in opposite directions for durative count items, compared to punctive count and durative mass items: whereas the ditransitive construction resulted in more “shortest” categorizations for punctive count and durative mass items, this effect was absent (numerically: reversed) for the durative count items.

#### Discussion of Experiment 2

This study gave participants the opportunity to estimate event durations using predefined time bins as choices, which might have been easier for them than coming up with time estimates on their own. The answer options in Experiment 2 were based on each item pair’s averaged estimates from Experiment 1a, which, as we discussed above, seemed fairly high. Nevertheless, we found significant differences for punctive count events: When presented in ditransitive light verb constructions (*give a kiss*), they were estimated to last less time than in transitive syntax (*kiss*). For durative count and durative mass events (*talking/giving a talk, advising/giving advice*), we did not observe such a difference, although the *β*-estimates indicate that durative mass events follow the same trend as punctive count events, with ditransitive structures pushing categorizations towards shorter bins.

Interestingly, the largest proportion of choices in both grammatical constructions and all three event types fell to the short orshortest options. Given that the choice options were based on previously obtained quartiles, the answers should have been roughly equally distributed. Thus, our data contribute to the literature on over- and underestimation of event duration in an interesting way: Open guesses as in Experiment 1a and 1b tend to overestimate (at least for the event types we used here, which, unlike in classical studies on the planning fallacy, did not include unpleasant chore-like events), whereas predefined categorizations are closer to more realistic event durations.^6^

As stated above, though, we were not *per se* interested in how long events are estimated to take, but in the influence of mass and count syntax on estimated event durations. Crucially, as in Experiment 1a and 1b, we found that count syntax shortens the time estimate for punctive, but not durative events. We had predicted this pattern because punctive verbs are often interpreted iteratively, and by using count syntax, one picks out one particular subevent. For durative verbs with count syntax, however, there is no distinct subevent to be picked out: Thus, we hypothesized that durative verbs might undergo a larger conceptual shift than when put in count syntax (*to give a talk*) than do punctive count or durative mass verbs.

The remaining studies test these possible mechanisms of how temporal estimates are affected by syntax: Experiments 3a and 3b investigate whether count syntax serves to single out one particular instance of an event. This could explain why events are imagined to be shorter in punctive count syntax. It would not, however, explain why durative count time-estimates were unaffected by changes in syntactic structure; in this case, we hypothesized that the lack of an effect in this condition is due to a conceptual shift between transitive verbs and count syntax, similar to the change from “iron” to “an iron”. This conceptual shift would be orthogonal to event duration. We investigated the existence of such a conceptual shift in Experiment 4.

## Experiment 3a: Event repetition

This section presents two studies that establish whether using count or mass syntax affects how many times an event is understood as occurring.

The theoretically most crucial prediction applies to punctive events. Punctive events, like kissing, are often understood iteratively, even if their lexical semantics merely conveys one single, telic, event (Kim & Kaiser, 2015). For example, people might interpret *John kissed Mary* as him kissing her more than once. However, when presented in count syntax (*to give a kiss*), only one kiss should be singled out. This would explain the consistently lower time estimates obtained in Experiments 1a, 1b, and 2.

Obviously, in order to be counted, a given event needs to be individuated, and we expect individuation to be easier in count than in mass syntax (Barner et al., 2008). So, for durative events in mass syntax, predictions are not quite as straightforward: *The professor advised her student* consists of many individual advising situations. The telic verb *give* in *The professor gave advice to her student* introduces a boundary to the advising event (Krifka, 1992); however, no definite article aids in the event individuation, so we might expect a weaker effect than for the punctive count events.

However, for durative events that can enter count syntax, we might predict the same trend as for punctive count events: *talking could convey more events than giving a talk*. Note that this prediction is independent of our second prediction, namely that there is a conceptual shift involved from a transitive durative verb to the same lexical item in ditransitive count syntax; and that any changes in event repetition counts would still be orthogonal to changes in event duration.

### Methods

#### Participants

For this study, we recruited 80 self-described English native speakers from Amazon Mechanical Turk.

#### Procedure

Participants read each sentence and then noted how many events they imagined reading the sentence. The exact instructions can be found under https://github.com/ewittenberg/QuickKissing.

#### Stimuli

We used the stimuli described in Table 1, counterbalanced across two lists.

#### Results

Our data spanned several orders of magnitude, (e.g., responses for punctive events in transitive frame ranged from 1 to 60), effects of construction are likely to operate proportional to intrinsic construal of numbers of events, as was the case with construed event durations in Experiments 1a and 1b. Therefore we log-transformed event-count responses for purposes of data summarization and analysis.

**Fig. 5.**
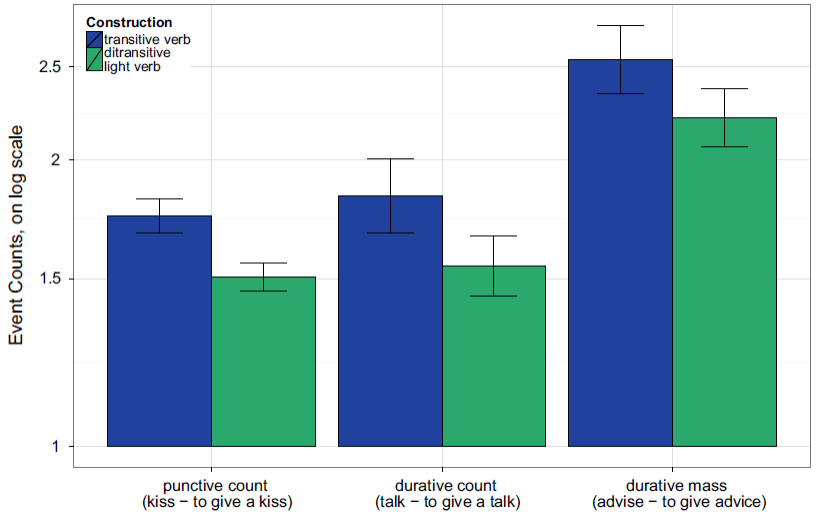
Count of imagined events in Experiment 3a. The y-axis is represented in log scale, but labeled with back-transformed counts (log-averages) for convenience.

Mean count of events was lower for ditransitive light verb constructions in each event category. Fig. 5 shows the pattern of results, with log event counts back-transformed to raw event counts for convenience of interpretation: For punctive count events, using a ditransitive light verb construction instead of the transitive verb reduced the mean count from 1.7 to 1.5 (log-averages). These effects were also numerically present for durative count and durative mass items, but variances in these constructions were higher.

Statistical analysis followed the same procedures as in Experiment 1a. The upper part of Table 5 shows results of the 3 × 2 ANOVA-style analyses (here, all random correlation parameters were removed to ensure model convergence). We find that construction had a significant main effect on event counts, event category was marginally significant, and their interaction was not. The lower half of Table 5 shows results of the planned tests of the simple effect of construction within each event category (random correlation parameters did not need to be removed). We see a significant effect of construction in punctive count pairs, but not in durative count or durative mass pairs.

#### Replication: Experiment 3b

**Fig. 6.**
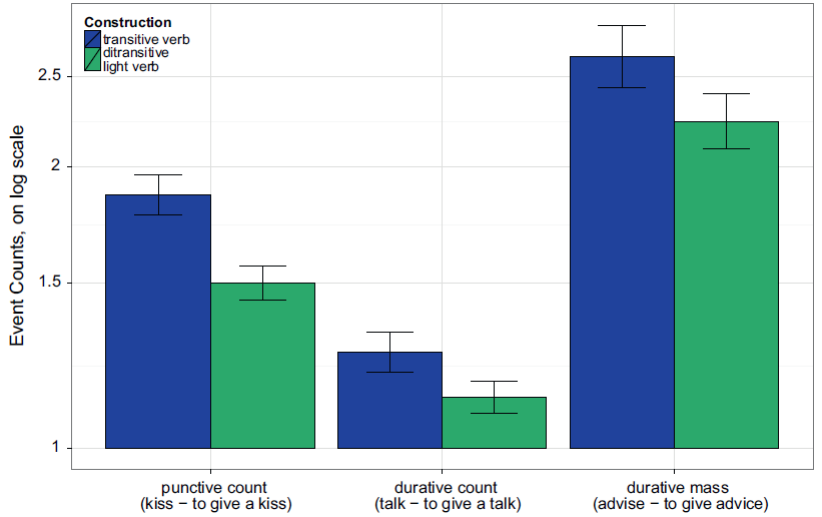
Count of imagined events in Experiment 3b. The y-axis is represented in log scale, but labeled with back-transformed counts (log-averages) for convenience.

We replicated this study by asking 80 native speakers on Amazon Mechanical Turk for the number of specific event types imagined after each sentence, for example “How many kisses did you just imagine?”, “How many talks did you just imagine?”, or “How many advices did you just imagine?” (instead of, as in Experiment 3a, asking the generic “How many events did you just imagine?”). Note that predictions for durative count items might be stronger in this study than in Experiment 3a, since we asked for the specific event (e.g., talks), thus excluding any super- or subevents people might have imagined as well, such as “climbing the podium”, “adjusting the microphone”, etc. We obtained a similar pattern of results (see Fig. 6).

Data analysis procedures were identical to those in Experiment 3a. Table 6 displays the results of the event count estimation test for the main effects, which were both significant, and their interaction, which was not. Pairwise comparisons (bottom part of Table 6) show main effects of construction for both punctive count and durative count, but not for durative mass events.

#### Discussion of Experiment 3a and 3b

This study investigated whether phrasing an event with mass or count syntax, instead of with a transitive verb, affects any iterative readings that are present.

Crucially, we see a significant reduction in imagined number of events as a measure of implicit iterativity from transitive verb encoding to ditransitive light verb encoding – but, as predicted, only consistently in punctive count events, and to a lesser degree, in durative count events. Thus, the more pronounced effect for durative count items in Experiment 3b, and the overall reduction in event counts in Experiment 3b, confirm our hypothesis that count syntax encourages individuating over subevents, leading to shorter event conceptualization.

Contrary to what the previous literature has claimed, durative events resulted in iterative readings, as well; we attribute that to the fact that events like *advising* or *talking*, while not easily segmentable by pieces of advice or specific identifiable talking events, do carry an element of interactivity: There is a back-and-forth between the advisor or talker, and the addressee; and there might also be habitual interpretations available. Thus, it could be that our participants conceptualized the “number of events” question as “number of subevents” – which would explain the occasional “more than one” answer for count syntax like *give a talk*.

However, we had hypothesized that count syntax might encourage a conceptual shift in durative verbs that acts orthogonally to any changes in temporal conceptualization. The last experiment investigates this possibility.

## Experiment 4: Event similarity

This study tested another prediction of our theory: that durative events in count syntax (*to give a talk*) should be conceptually further apart from their transitive verb counterparts (*to talk*) than punctive events in count syntax, or durative events in mass syntax. This prediction is drawn from the analogy to mass nouns, such as *glass* or *iron* into count syntax: In many cases, the count noun denotes objects that are conceptually related, yet different, from the noun in mass syntax, such as in *a glass or an iron*. If the durative events in count syntax behave in parallel, we should expect them to be conceptually further apart from their transitive counterparts than punctive events in count or durative events in mass syntax.

### Methods

#### Participants

We recruited 40 self-described English native speakers from Amazon Mechanical Turk for this study.

#### Procedure

We asked participants to rate event similarity between transitive and ditransitive frames on a 7-point Likert scale where 1 indicated “same event”, and 7, “completely different event”. The exact instructions can be found under https://github.com/ewittenberg/QuickKissing.

#### Stimuli

We used the 20 item pairs shown in Table 1, without the sentence context. In addition, we created 26 filler pairs that ranged from very close synonyms (*repairing – fixing*) to very different events (*working* – *being lazy*).

#### Results

For filler items, the average rating was 3.62 (*SD* = 2.1), with the full range of the scale being used.

**Fig. 7.**
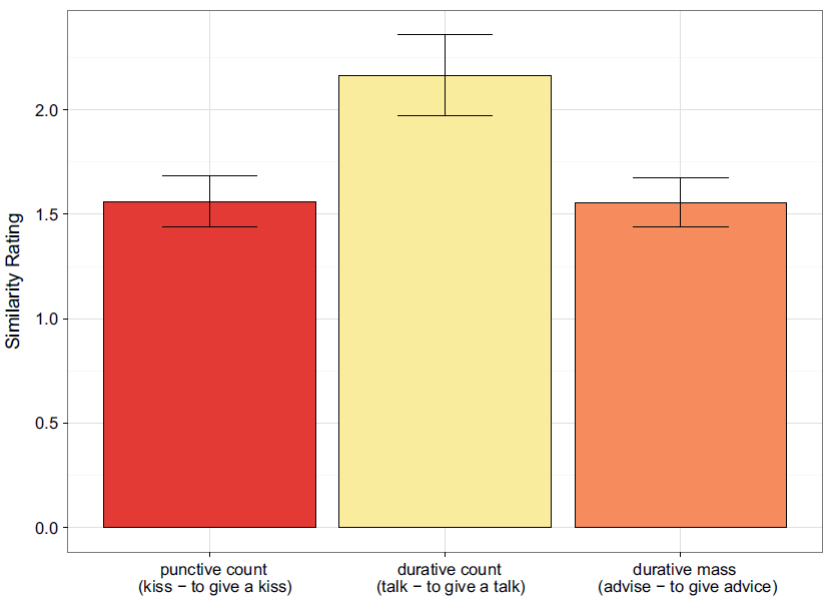
Experiment 4: Punctive count and durative mass item pairs were rated more similar to each other than durative count item pairs.

Fig. 7 shows the rating results for critical pairs. Punctive events (*to kiss vs to give a kiss*) received a mean rating of 1.6 (*SD* = 1.0). Pairs of durative verbs and durative verbs in mass syntax (*to advise vs to give advice*) received a similarly low rating (mean: 1.5, *SD* = .9). This means that for both punctive count and durative mass items, both constructions denote very similar events. As predicted, difference ratings for pairs of durative verbs and durative verbs in count syntax (*to talk vs to give a talk*) were higher: 2.1 (*SD* = 1.5).

Like in Experiments 1a, 1b, and 3, we used linear mixed models with maximal random effects structure (by- subjects intercepts and event-category slopes, by-items intercepts; no random correlation parameters needed to be removed). Table 7 displays the results from 3-level omnibus ANOVA-style analysis, as well as simple comparisons between all pairs of event categories. There was a significant main effect of event category in the omnibus analysis. The pairwise comparisons show no significant difference between similarity ratings for punctive count and durative mass event pairs; durative count pairs, on the other hand, were rated significantly less similar to each other than pairs in the other two event categories.

#### Discussion of Experiment 4

Experiment 4 confirms the intuition that while *giving a kiss and kissing*, as well as *giving advice* and *advising*, belong ontologically to the same event, *giving a talk* and *talking* are conceptually further apart – albeit with considerable overlap. This result contributes to claims made in the literature that light verb constructions often help to close lexical gaps (Glatz, 2006; Grimshaw & Mester, 1988; Miyagawa, 1989), and it draws our attention to interesting parallels to using count syntax on mass nouns (Gordon, 1985; Srinivasan & Rabagliati, 2015; Wiese & Maling, 2005).

These results are crucial to our question of how using mass or count syntax affects the construal of event duration: If a durative lexeme in count syntax covers a conceptually different event than the same lexical item in a transitive verb frame, the conceptualization of event duration applies to the new concept, and thus any changes in duration estimates would be largely coincidental.

## General discussion

We have presented a family of experiments that tested the hypothesis that describing an event with mass versus count syntax affects the construal of event similarity and duration in a way that is systematically predictable from the interaction of mass versus count syntax and verb semantics (see Fig. 1). These predictions were built on insights from formal semantics that has pointed out similarities between mass nouns and durative, non-atomic events on the one side, and count nouns and punctive, atomic events on the other side (Bach, 1986; Casati & Varzi, 2008; Harley, 2005; Jackendoff, 1991; Krifka, 1992; Wellwood et al., 2016).

Specifically, we had predicted that punctive events in count syntax (*give a kiss*) are construed as taking less time than in transitive verb frame (*kiss*); this pattern was predicted because in punctive events, which are often interpreted iteratively (Kim & Kaiser, 2015), one atomic-subevent is singled out by count syntax.

In durative events, the same pattern was predicted for events in mass syntax, such as *giving advice*. In combina tion with the telic verb give, the mass noun carves out a limited portion of the event structure, leading to shorter event conceptualization.

A different pattern was predicted for durative verbs in count syntax (*give a talk versus talk*): We expected durative events in count syntax to be semantically further from their verbal counterparts (*to talk – a talk*) than punctive events (*to kiss – a kiss*). Since these shifts were presumably orthogonal to the temporal structure of the event, we did not make any predictions about duration estimates. These predictions stemmed from insights about the parallels between durative events and mass nouns: When some mass nouns are forced into count syntax (*a glass; an iron*), there is a semantic shift from the mass noun meaning (*glass; iron*).

Our experimental results broadly confirm these predictions. In Experiments 1a and 1b, we elicited open duration estimates, which were consistently lower for punctive events in count syntax and durative events in mass syntax than when they occurred in transitive frames; durative events in count syntax did not show this effect. Experiment 2 replicated these results by using the quartiles of each event’s individual duration estimates obtained in Experiment 1a as answer choices; again, punctive count and durative mass ditransitive structures were judged to take less time than transitive structures, while there was no difference for durative events in count syntax.

On our theory, a key factor for the reduction of duration estimates in punctive verbs was that people should imagine fewer events taking place than in the ditransitive count frame, due to the combination of a telic verb (*give*) and nominal count syntax (*a kiss*). However, this effect should be weaker for durative events in either count or mass syntax when participants are asked for number of events. Experiment 3a confirmed these predictions. Experiment 3b showed that when people are asked for how many specific events they imagined – how many talks, for example – the reduction in event counts is significant even for durative events in count syntax. This confirms both the observation that event individuation is easier in count syntax (Barner et al., 2008), and that durative verbs behave similarly to mass nouns (Krifka, 1992).

Experiment 4 showed, consistent with our predictions, that durative events undergo a semantic shift in count syntax: The semantic differences between transitive and ditransitive frames were larger in durative count pairs (*talk – give a talk*) than in punctive-count pairs (*kiss – give a kiss*) or durative-mass pairs (*advise – give advice*).

Thus, the shift from a transitive to a ditransitive frame has systematically predictable repercussions, depending on whether the event type was durative or punctive, and depending on whether the event was described with a mass or with a count noun.

These results provide psycholinguistic evidence for the observation in formal semantics that reference properties of syntactic objects change the reference properties of the whole predicate (Krifka (1992); also see Quine (1969),erkuyl (1972)). In our case, the nominalization of eventive verbs using light verb constructions with a telic verb helped to divide experience into countable units (for punctive verbs in count syntax and durative verbs in mass syntax), or introduced a semantic shift (for durative verbs in count syntax), similar to what happens with mass nouns in count syntax.

Our results also complement studies that suggest that people conceptualize events differently depending on subtle choices among syntactic alternations (Fausey & Boroditsky, 2010; Johnson & Goldberg, 2013; Wittenberg & Snedeker, 2014) and that conversely, subtle changes in event structure result in changing preferences between syntactic alternations (Gropen et al., 1991). This growing family of experiments, together with a number of corpus studies (Benor & Levy, 2006; Bresnan, Cueni, Nikitina, & Baayen, 2007), contributes to our understanding how syntactic and semantic structures, as well as processing pressures, such as a preference for using frequent, accessible lexical items, lead to a speaker’s decision between two seemingly equivalent constructions.

These findings may also help interpret results from less explicit tasks. We know from behavioral, ERP, and MEG studies that light verb constructions are processed differently from non-light constructions (Briem et al., 2009; Piñango, Mack, & Jackendoff, 2006; Wittenberg & Piñango, 2011; Wittenberg, Paczynski, Wiese, Jackendoff, & Kuperberg, 2014). So far, however, only semantic role mismatches had been identified as a factor contributing to the processing difference (Wittenberg et al., submitted for publication; Wittenberg & Snedeker, 2014). Based on the present work, we might hypothesize that the calculation of temporal structure also plays a role in the real-time processing of light verb constructions.

A question this article raises is how well these results might generalize to other constructions, for example, other light verb constructions with atelic verbs. We would predict that other light verb constructions with bounded verbs would lead to the same effect; for example, *John took a shower* should be estimated as taking less time than *John showered*, whereas *John had a shower* should affect estimated event durations to a lesser degree, since both verbs (*to shower and to have*) are atelic. Another area where the interaction of count/mass syntax and verbal aspect might have repercussions for the temporal conceptualization of events are idioms. In the literature on idiom comprehension there is a consensus that the lexical items in an idiomatic phrase such as *to kick the bucket* are accessed individually as well as holistically (Cutting & Bock, 1997; Holsinger, 2013; Sprenger, Levelt, & Kempen, 2006; Tabossi, Wolf, & Koterle, 2009; Titone & Libben, 2014). So if an idiom consists of a semelfactive verb (*kick*) and a count noun (*bucket*), and its semantically transparent counterpart is a semantic achievement (*to die*) we would predict that *John kicked the bucket* will be estimated to take a shorter time than *John died*. These predictions remain to be addressed in future studies.

In sum, this article showed that using mass versus count syntax affects the construal of event similarity and duration in a way that is systematically predictable from the interaction of mass versus count syntax and verb semantics. Our results confirm observations from formal semantics that the properties of objects and events are mirrored in count/mass syntax and verbal aspect, and are advancing our understanding of the effects of syntactic choices on subtle aspects of event construal.

## Figures and Tables

**Fig. 8.**
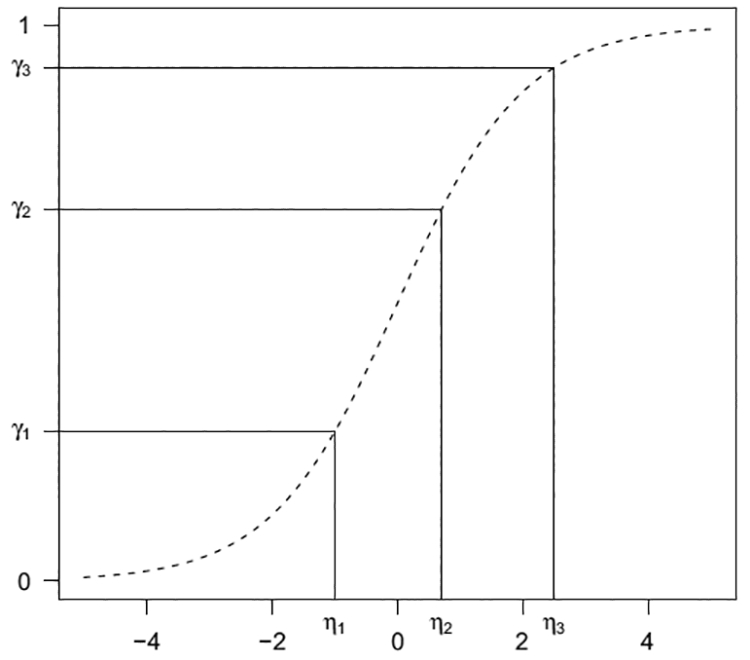
Cumulative logit models. The *N* − 1 linear predictors {*n*_*j*_} induce a set of *N* multinomial response category probabilities through the inverse logit transform.

**Table 1:** Experimental item pairs used for all experiments.

	Count	Mass
Punctive	to kiss – to give a kiss	
	*to embrace – to give an embrace*	
	*to hug – to give a hug*	
	*to kick – to give a kick*	
	*to poke – to give a poke*	
	*to shake – to give a shake*	
	*to cuddle – to give a cuddle*	
Durative	*to talk – to give a talk*	*to advise – to give advice*
	*to address – to give an address*	*to thank – to give thanks*
	*to lecture – to give a lecture*	*to assure – to give assurance*
	*to present – to give a presentation*	*to encourage – to give encouragement*
	*to speak – to give a speech*	*to recognize – to give recognition*
	*to scold – to give a scolding*	*to support – to give support*
		*to assist – to give assistance*

**Table 2:** Table of likelihood estimation results for duration estimates in Experiment 1a, testing the main effects and their interaction (upper part), and the results of testing the main effect of construction in planned pairwise comparisons (lower part).

	*Df*	*χ*^2^	*p*-value
Construction	1	7.9	.005 ***
Event category	2	23.02	.000 ***
Construction × event category	2	3.28	.196 *n.s*.
Punctive count − construction	1	3.54	.059 .
Durative count − construction	1	0.15	.690 *n.s*.
Durative mass − construction	1	6.17	.013*

Asterisk = significant at the 5% level;. = significant at the 10% level; n.s. = not significant.

**Table 3:** Table of likelihood estimation results for duration estimates in Experiment 1b, testing the main effects and their interaction (upper part), and the results of testing the main effect of construction in pairwise comparisons (lower part).

	*Df*	*χ*^2^	*p*-value
Construction	1	4.98	.026 *
Event category	2	27.52	<.0001 ***
Construction × event category	2	6.10	.047 *
Punctive count − construction	1	9.64	.026 **
Durative count − construction	1	0.40	.531 *n.s*.
Durative mass − construction	1	0.95	.330 *n.s*.

Asterisk = significant at the 5% level;. = significant at the 10% level; n.s. = not significant.

**Table 4:** Experiment 2: Regression table for categorizations.

	*β*	SE		
Shortest|short	−0.114	0.256		
Short|long	2.201	0.265		
Long|longest	4.669	0.320		
	*Df*	*β*	LR.stat	*p*-value
Construction	1	−0.195	4.079	.043 *
Event category	2		4.46	.107 n.s.
Construction × event category	2		5.21	.070 .
Punctive count − construction	1	−0.811	4.995	.025 *
Durative count − construction	1	0.290	1.263	.262 *n.s*.
Durative mass − construction	1	−0.607	2.156	.142 *n.s*.

Asterisk = significant at the 5% level;. = significant at the 10% level; n.s. = not significant.

**Table 5:** Table of likelihood estimation results for event counts in Experiment 3a, testing the main effects and their interaction (upper part), and pairwise comparisons (lower part).

	*Df*	*χ*^2^	*p*-value
Construction	1	7.011	.008 **
Event category	2	5.326	.069 .
Construction × event category	2	0.016	.992 n.s.
Punctive count − construction	1	7.187	.007 **
Durative count − construction	1	1.086	.297 *n.s*.
Durative mass − construction	1	1.030	.310 *n.s*.

Asterisk = significant at the 5% level;. = significant at the 10% level; n.s. = not significant.

**Table 6:** Table of likelihood estimation results for event counts in Experiment 3b, testing the main effects and their interaction (upper part), and pairwise comparisons (lower part).

	*Df*	*χ*^2^	*p*-value
Construction	1	11.034	.000 ***
Event category	2	8.297	.016 *
Construction × event category	2	1.001	.606 *n.s*.
Punctive count − construction	1	5.391	.020 *
Durative count − construction	1	4.063	.043 *
Durative mass − construction	1	2.234	.135 *n.s*.

Asterisk = significant at the 5% level; . = significant at the 10% level; n.s. = not significant.

**Table 7:** Experiment 4: Table of likelihood estimation results for event similarity in Experiment 4, testing the main effect of event category (upper part), and pairwise comparisons between event categories (lower part).

	*Df*	*χ*^2^	*p*-value
Event category	2	6.819	.033 *
Punctive count vs durative count	1	3.799	.046 *
Durative count vs durative mass	1	4.869	.027 *
Punctive count vs durative mass	1	0.000	.984 *n.s*.

Asterisk = significant at the 5% level; . = significant at the 10% level; n.s. = not significant.

## Footnotes

1 Telic events are said to involve some kind of natural endpoint (Andersson, 1972; Bauer, 1970; Garey, 1957; Klein, 1994; Vendler, 1957, among others). This definition covers accomplishments, *like to draw a circle*, but not all punctive events. The classical definition of punctive events is that they only take a moment in time. How long this moment takes, however, is underspecified: The duration of to *sneeze* depends entirely on the sneezer; *to explode* can conceptually take more than a few seconds; and the event described by *to break* might last for a few minutes. Thus, temporal properties make up one part of the diagnostics; the other part is contributed by the intuition that there is a natural endpoint to a given event.

2 Another option would be a strictly linear model: A lexical item is inserted into a syntactic tree, its phonological form created, and its meaning read off by the comprehender, without any feedback loop to the semantic or conceptual system. In this case, both forms of the lexical items (e.g., *kiss, talk* or *advise* as either noun or verb) are ontologically linked to the same event structure regardless of grammatical status, and it should not make a difference for duration estimates whether they appear as a verb or in either mass or count noun syntax, since the comprehender’s lexicon is set up the same way – *kiss* conveys the same event regardless of whether it appears as verb or noun. Thus, this linear model would not predict any differences in duration estimates.

3 In fact, *talk* and *speak* do not occur in transitive frames, but rather with prepositional objects (*talk*_***_*(to) the students*). In the context of this article, however, we use “transitive” to mean two-place argument structures with either a Direct Object or a Prepositional Object.

4 The missing item in the durative count category was *condole* – *giving condolences*, which we ultimately decided to exclude because *condole* is used only very rarely as a verb in American English. In English, there are no punctive events in mass syntax with *give*.

5 We implemented this coding in R as numeric predictors rather than as factors, which allows us to test main effects in the presence of the interaction through likelihood-ratio tests; see Levy (2014), among others, for details.

6 We tested this explanation by giving participants shorter answer options that were not based on previous estimates. We found a much more equal distribution of answers. Since this study is tangential to the goal of this article, it is not reported in detail, but the data are accessible under https://github.com/ewittenberg/QuickKissing.

## Appendix A. Stimuli used for Experiments 1–3

Stimuli are shown in transitive frame. In the ditransitive light verb construction, the underlined verb would serve as the light noun, i.e., *After their first date, Douglas kissed Mary* → After their first date, Douglas gave a kiss to Mary.

1. Punctive Count
  - After their first date, Douglas kissed Mary.
  - Laughing nastily, the thug kicked the victim.
  - When they met up, Nathasha hugged Cynthia.
  - Noam embraced Jennifer before they split up.
  - Julius cuddled his brother at bedtime.
  - Laura poked Owen because he was so annoying.
  - Martin shook the cocktail before he served it.
2. Durative Count
  - The professor lectured on injustice yesterday.
  - The CEO talked about his latest sales strategy last night.
  - The President spoke about affordable education on Wednesday.
  - During the community art show, the composer presented his latest work.
  - After the graduation ceremony, Tom Hanks addressed the students.
  - The mother scolded the child today.
3. Durative Mass
  - The professor advised her student on his paper yesterday.
  - Yesterday, the nurse assisted Dr. Kohler in the emergency room.
  - After the job interview, Sheila assured Keith.
  - Before Kelly left for a year abroad, her friends encouraged her.
  - After the devastating flood, the mayor recognized the rescue workers.
  - His new girlfriend supported Sam after his divorce.
  - At the summer party, the university official thankedthe academic community for their efforts.

## Appendix B. The mixed-effects cumulative logit model used for Experiment 2

Data were analyzed using R (R Core Team, 2014), specifically with mixed-effects cumulative logit model using the ordinal package (Christensen, 2015). For ordered response categories 1; 2;…; *N*, a mixed-effects cumulative logit model specifies response probabilities for a given datum i as a function of predictor variables as follows. There are *N* − 1 linear predictors, each of the following form:

$$\eta_{ij}=\alpha_{j}+\sum_{k}\beta_{k}x_{ik}+\sum_{k}b_{k}z_{ik}\;j\in \left\{1,2,\ldots ,N-1\right\}$$

where the {*x*_*ik*_} and {*z*_*ik*_} are fixed- and random-effects predictors respectively, the {*β*_*k*_} and {*b*_*k*_} are fixed- and random-effects regression parameters respectively, and *α*_*i*_ is a THRESHOLD PARAMETER for the boundary between the j-th and (*j* +1)-th response categories. (The threshold parameters play a role analogous to the intercept in an ordinary mixed logit model.) The linear predictors are related to cumulative probabilities {*γ*_*ij*_} through the inverse logit function:

$$\gamma_{ij}=P\left(Y_{i}\leq j\right)=\frac{e^{\eta_{ij}}}{1+e^{\eta_{ij}}}$$

The probability of datum i having response category *j* is thus

$$P\left(Y_{i}=j\right)=\{\begin{array}{ll} \gamma_{i1} & j=1\\ \gamma_{ij}-\gamma_{ij-1} & j\in \left\{2,\ldots ,N-1\right\}\\ 1-\gamma_{ij-1} & j=N \end{array}$$

That is, the {*γ*_*ij*_} carve up the interval [0, 1] into a set of category response probabilities, as illustrated in Fig. 8. In mixed-effects cumulative logit models, the random-effects regression parameters are assumed to be drawn from some multivariate normal distribution, the covariance matrix of which is estimated jointly along with the threshold parameters and fixed-effects regression parameters (here, via Laplace-approximated maximum likelihood).
